# Using cluster analysis to reconstruct dengue exposure patterns from cross-sectional serological studies in Singapore

**DOI:** 10.1186/s13071-020-3898-5

**Published:** 2020-01-17

**Authors:** Sorawat Sangkaew, Li Kiang Tan, Lee Ching Ng, Neil M. Ferguson, Ilaria Dorigatti

**Affiliations:** 1 Department of Infectious Disease, Faculty of Medicine, Imperial College London, London, UK; 2 Environmental Health Institute, National Environment Agency, Singapore, Singapore; 3 School of Biological Sciences, Nanyang Technological University, Singapore, Singapore; 4 MRC Centre for Global Infectious Disease Analysis, School of Public Health, Imperial College London, London, UK

**Keywords:** Dengue exposures, Cluster analysis, Serological survey

## Abstract

**Background:**

Dengue is a mosquito-borne viral disease caused by one of four serotypes (DENV1-4). Infection provides long-term homologous immunity against reinfection with the same serotype. Plaque reduction neutralization test (PRNT) is the gold standard to assess serotype-specific antibody levels. We analysed serotype-specific antibody levels obtained by PRNT in two serological surveys conducted in Singapore in 2009 and 2013 using cluster analysis, a machine learning technique that was used to identify the most common histories of DENV exposure.

**Methods:**

We explored the use of five distinct clustering methods (i.e. agglomerative hierarchical, divisive hierarchical, K-means, K-medoids and model-based clustering) with varying number (from 4 to 10) of clusters for each method. Weighted rank aggregation, an evaluating technique for a set of internal validity metrics, was adopted to determine the optimal algorithm, comprising the optimal clustering method and the optimal number of clusters.

**Results:**

The K-means algorithm with six clusters was selected as the algorithm with the highest weighted rank aggregation. The six clusters were characterised by (i) dominant DENV2 PRNT titres; (ii) co-dominant DENV1 and DENV2 titres with average DENV2 titre > average DENV1 titre; (iii) co-dominant DENV1 and DENV2 titres with average DENV1 titre > average DENV2 titre; (iv) low PRNT titres against DENV1-4; (v) intermediate PRNT titres against DENV1-4; and (vi) dominant DENV1-3 titres. Analyses of the relative size and age-stratification of the clusters by year of sample collection and the application of cluster analysis to the 2009 and 2013 datasets considered separately revealed the epidemic circulation of DENV2 and DENV3 between 2009 and 2013.

**Conclusion:**

Cluster analysis is an unsupervised machine learning technique that can be applied to analyse PRNT antibody titres (without pre-established cut-off thresholds to indicate protection) to explore common patterns of DENV infection and infer the likely history of dengue exposure in a population.

## Background

Dengue is a mosquito-borne viral disease that poses a high burden on public health worldwide. In a study in 2016, dengue infection was estimated to cost 8.9 billion US dollars per year and 12 disability-adjusted life years (DALYs) per 100,000 people [1]. A recent study estimated that more than half of the world’s population is at risk of dengue infection annually [2]. Of those, 390 million people are infected and 21,000 people die from dengue each year.

Dengue virus (DENV) has four serotypes (DENV-1 to DENV-4) and humans acquire dengue disease through infected mosquito bites. Most dengue infected individuals are asymptomatic and dengue disease is often self-limiting. However, some individuals infected with DENV can develop severe and life-threatening conditions [2, 3]. Following a dengue infection, short-term heterologous immunity against all serotypes and long-term homologous immunity against the infecting serotype are mounted [4]. Epidemiological evidence suggests that secondary infections are more frequently associated with severe disease, with the leading hypothesis for this phenomenon being antibody-dependent enhancement (ADE), whereby antibodies elicited against the primary-infecting strain enhance infection by a secondary heterologous strain [5, 6]. According to the World Health Organization recommendations [3, 7], the plaque reduction neutralisation test (PRNT) is the gold standard assay for detecting serotype-specific antibody levels. In the test, sample sera are mixed with progeny virus and animal cells before being overlaid with semi-solid media. The areas of viral infected cells (plaques) are counted and compared with a control sample (without antibodies) to determine the percent reduction [8, 9]. The effective dose of antisera reducing the number of viral plaques of a control sample by 50% is reported as PRNT_50_, which is a measure of the levels of neutralising antibodies.

Neutralising serotype-specific antibody levels could reflect the history of dengue exposure. Recent studies have demonstrated that primary and post-primary dengue infections show different neutralising antibody level patterns which change dynamically in time [10–12]. During the convalescent phase (1 week post-symptom onset) after primary infection, homologous and heterologous antibodies are typically present at low detection levels. These levels then considerably increase over 6–12 months and then both heterologous and homologous antibody levels keep increasing at the same rate during year one to year two after infection [10, 11]. In post-primary infections, both heterologous and homologous antibody levels rise at the same rate during the convalescent phase and then dramatically decrease over 6 months after infection [10, 11]. Heterologous antibodies have been demonstrated to decay faster than homologous antibodies. In addition, the PRNT titres of both homologous and heterologous antibodies in post-primary infections are generally higher than the titres observed in primary infections over the first year after infection [10, 11]. Clapham et al. [11] have shown that after the 6-month period post-infection, neutralising antibodies levels remain stable for 2–3 years, after which time the antibody levels typically decay.

Cluster analysis is an unsupervised machine learning technique used to classify objects into discrete groups, which have high similarity within the membership group and low similarity with other groups. This technique does not rely on any prior classification based, for instance, on cut-off thresholds. The (dis)similarity of different objects is evaluated using the concept of distance measurements among objects, where multiple measures have been proposed depending on the nature of the problem analysed. Cluster analysis has been increasingly applied in health science research in recent years to investigate exposure risks, diagnosis and treatment [13].

Serological prevalence surveys for dengue (as well as other infectious diseases) have been conducted to assess the levels of immunity in a population, using pre-defined cut-off values to classify individual-level antibody levels into a positive or negative category to ultimately provide an aggregated estimate of the proportion of seropositive population. Here, we present an analysis of the individual-level PRNT data collected in two serological surveys conducted in Singapore to identify the most common patterns of dengue antibody levels and infer the most likely histories of dengue infection. Cluster analysis was used to classify dengue seropositive subjects into groups (or clusters) according to their individual-level PRNT_50_ data collected in two seroprevalence surveys conducted in Singapore in 2009 and 2013. The results presented in this study provide nuanced estimates of population immunity, which can help public health policy makers evaluate outbreak risks, containment and control planning.

## Methods

### Data

We analysed the PRNT_50_ titres of 509 seropositive individuals obtained from two cross-sectional seroprevalence surveys conducted in Singapore in 2009 and 2013. A brief overview of the data collection process is provided below, with refined details available in [14]. Residual blood samples of healthy adults were drawn from blood donors by the Blood Service Group, Health Science Authority in 2009 and 2013. Of approximately 12,000 blood samples collected in each survey, 3,995 were randomly sampled based on an estimate of dengue seroprevalence at 59% with 99% confidence and 2% precision. The samples were then screened for dengue IgM and IgG antibodies by Panbio Dengue IgM capture ELISA and IgG ELISA (Alere Inc., Waltham, MA, USA). Among those with positive results (defined as having > 11 Panbio units), 30 samples were randomly selected in each age-group (16–20, 21–25, 26–30, 31–35, 36–40 and 56–60 years) for PRNT testing. The PRNT assay used two local viral strains for each dengue serotype as detailed in Additional file 1: Table S1.

### Cluster analysis

We chose to retain the average of the PRNT_50_ titres of the two viral strains against the same serotype to avoid variable redundancy due to the high correlation of titres between the same serotypes (Additional file 2: Figure S1). The PRNT_50_ titres that were coded as less than 10 (“< 10”) and more than 1000 (“> 1000”) were replaced by 5 and 2000, respectively. All PRNT_50_ titres were log-transformed (base 10) to reflect the natural scale of the dilution assay and the assay’s variability [15].

We clustered the 509 seropositive PRNT_50_ profiles using two nested clustering methods (agglomerative hierarchical and hierarchical divisive clustering), two partitioning clustering methods (K-means and K-medoids clustering) and one model-based clustering method. The agglomerative hierarchical clustering method initially assumes that each single data point forms a cluster and then iteratively nests the most similar clusters together. In contrast, the divisive hierarchical clustering method assumes that all data points are initially contained in a single cluster and then the most dissimilar data points are iteratively separated. In the K-means method the centres of the clusters were initially set guided by an agglomerative hierarchical algorithm, and the data points were assigned to the closest centres. Then iteratively, new centres were computed by minimising the total sum of squared errors (SSE) of distances between each data point and the closest centroid. This process was computed repeatedly until centroids were stable. The K-medoids algorithm is similar to the K-means algorithm but it minimizes the sum of dissimilarities between each data point and the data points labelled as centroids. Model-based clustering assumes that all variables are normally distributed and the dataset is a mixture of more than two component distributions. Each component (or cluster) is described by a probabilistic model through associated probability density functions. The model parameters were estimated using the Expectation Maximization (EM) algorithm and each data point was assigned to the component with the highest probability.

We used the Euclidean distance as metric for all clustering methods and tested each method on multiple numbers of clusters (from 4 to 10 clusters). Ward’s method was used within the agglomerative hierarchical clustering procedure.

### Cluster validation

In the absence of classification (e.g. the assignment of a dengue status according to the existing PRNT_50_ titres), we used three internal validation metrics (i.e. the Dunn index, silhouette width and adjusted connectivity) to identify the optimal clustering results. Dunn index is the ratio between the minimal distance between data points in different clusters. The values of this ratio ranges from zero to infinity, with higher values indicating better clustering results, reflecting larger separation between clusters and smaller separation between data points within the same cluster. Silhouette width is defined as the average silhouette values among all clusters, where the silhouette values are calculated as the mean distance within a cluster divided by the mean distance of the closest cluster. The values of silhouette width range from − 1 to 1, with values approaching 1 indicating better clustering results as intra-cluster distances are considerably smaller than inter-cluster distances. Adjusted connectivity defines the degree of connectedness among data points within the same cluster. Connectivity values range from 0 to infinity, with values close to 0 representing completely separated clusters.

We employed the R package *optCluster* (R version 3.4.3) to determine the optimal clustering method and the optimal number of clusters [16]. We implemented the five clustering methods (agglomerative hierarchical, hierarchical divisive, K-means, K-medoids and model-based clustering) with the number of clusters ranging between 4–10 and evaluated the clustering results using the Dunn index, silhouette width and adjusted connectivity as validating metrics. For each number of clusters analysed, we used weighted rank aggregation to generate a rank list of the clustering methods. The first ranked clustering method within the particular number of clusters analysed was considered the optimal method.

### Characterising immunity patterns

We statistically described the immunity patterns of the clusters obtained with the optimal clustering method using the average PRNT_50_ titres against the two strains of each dengue serotypes (Additional file 1: Table S1). We presented the results in terms of median, interquartile and range of the log transformed PRNT_50_ titres. We also described the proportion of population in each cluster.

### Sensitivity analysis

In a sensitivity analysis we explored the sensitivity of the results obtained on the aggregated data collected in 2009 and 2013 from the results obtained by analysing the PRNT_50_ titres collected in 2009 and 2013 separately. Sensitivity analysis was also conducted to test the robustness of the results obtained using the average PRNT_50_ titres for each serotype with the results obtained using the original 8 PRNT_50_ titres (two PRNT_50_ titres for each serotype), as described in Additional file 1: Table S1. Finally, we explored the effect that using the Gower distance (which is a distance measure defined for mixtures of continuous and categorical variables) in place of the Euclidean distance (which is only defined for continuous variables) had on the clustering results obtained on the aggregated dataset having added the year of sample collection (2009 or 2013) as a categorical variable.

## Results

We applied the five clustering algorithms (agglomerative hierarchical, divisive hierarchical, K-means, K-medoids and model-based clustering) with 4 to 10 clusters for each method. The four variables (i.e. the average PRNT_50_ titres of the two viral strains against each serotype) were used in place of the eight PRNT_50_ titres originally available to remove collinearity and variable redundancy (Additional file 2: Figure S1). The clustering results were then evaluated with the three internal validating metrics. We found that the K-means clustering algorithm with 6 clusters achieved the highest weighted rank aggregation and was thus considered the optimal clustering algorithm.

**Fig. 1:**
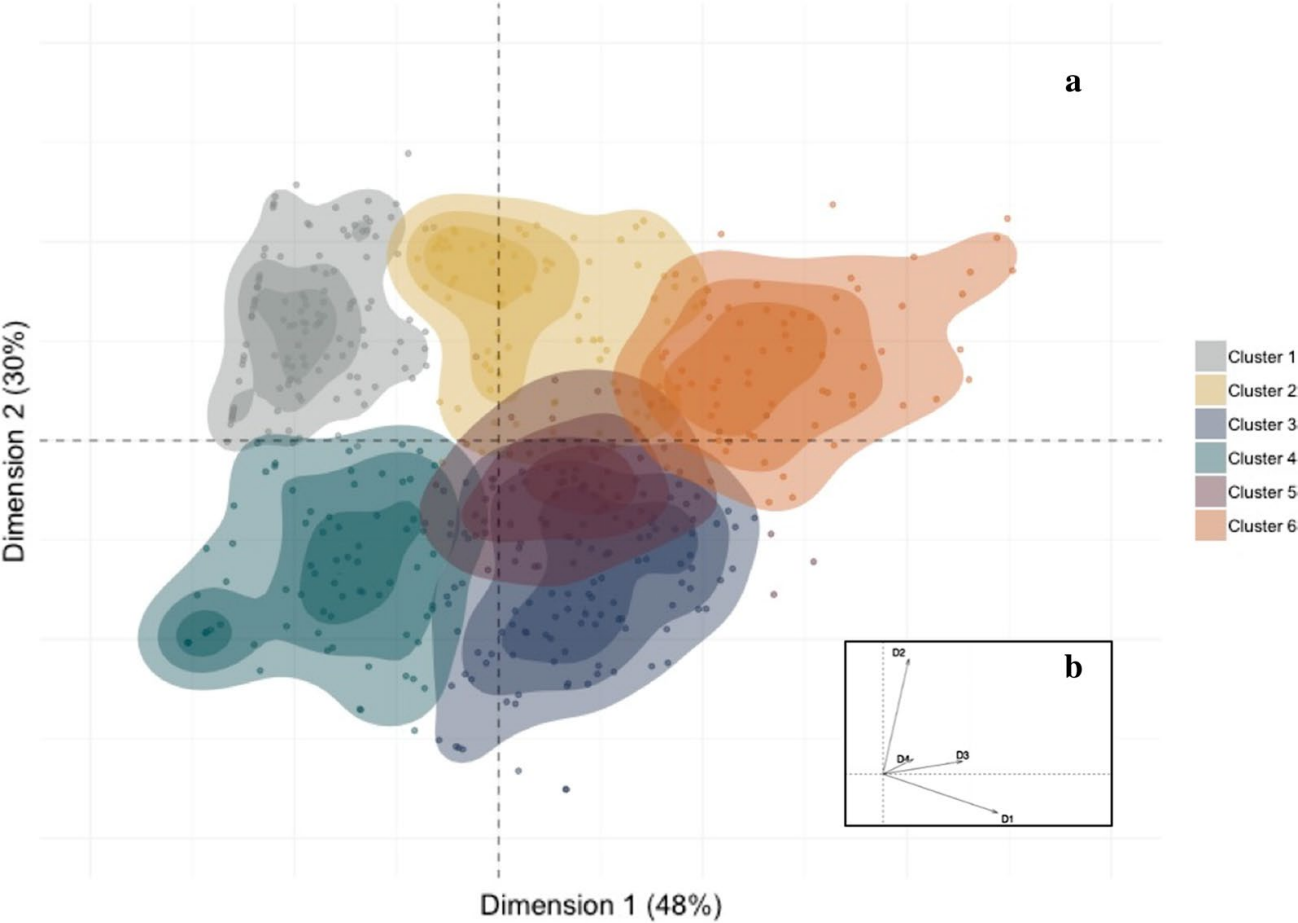
**a** The six clusters of 509 seropositive individuals obtained with the K-means algorithm. The x- and y-axes represent the two principle components from principle components analysis and account for 48% and 30% of the variance in the data, respectively. Colour shading represents the density of the data. **b** The four PRNT_50_ vectors used in the main analysis plotted in the two-dimensional principle component plane

The six clusters obtained using K-means algorithm are shown in Fig. 1a, where the location of the data points (each point represents one subject) is interpreted in relation to the four average PRNT_50_ titres used as variables in our analysis, which are shown as vectors in Fig. 1b. The subjects assigned to the same cluster are located close to each other, with the colour shading representing the density of the points in each cluster (darker colours represent more populated regions close to the centre of each cluster). The top five ranked clustering scenarios and their internal validation metrics are presented in Additional file 1: Table S2.

**Fig. 2:**
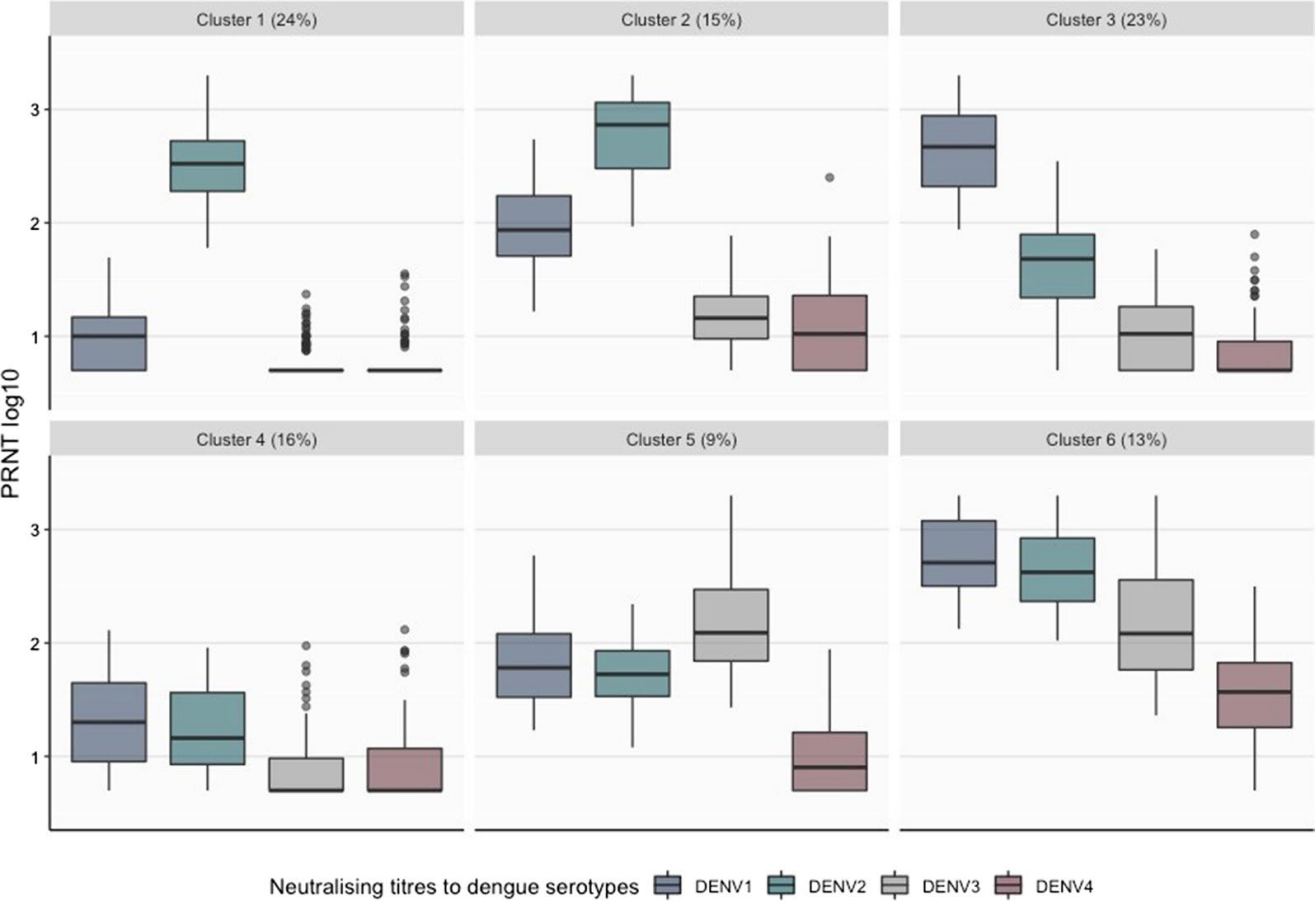
Median (bold line), interquantiles (box), range (vertical line) and outliers (points) of the log_10_ PRNT_50_ titres against DENV1-4 characterising the six clusters obtained with the K-means algorithm. The colours blue, grey, green and blown represent DENV1, DENV2, DENV3 and DENV4, respectively

Cluster-level statistics of the average PRNT_50_ antibody titres used as variables in the analysis are shown in Fig. 2. We found that 24% of the subjects enrolled in the 2009 and 2013 serological surveys were in cluster 1, which was characterised by DENV2 dominant titre. Cluster 2, accounting for 15% and cluster 3 accounting for 23% of the subjects were characterised by co-dominant titres against DENV1 and DENV2. However, the titre of DENV2 predominated in cluster 2 and the titre of DENV1 predominated in cluster 3. Low PRNT_50_ titres against all serotypes were observed in cluster 4, which accounted for 16% of the subjects in the seroprevalence studies. The remaining clusters, accounting for 9% (cluster 5) and 13% (cluster 6) of the subjects, displayed multitypic PRNT_50_ patterns with dominant titres against DENV3 and DENV1, respectively.

**Fig. 3:**
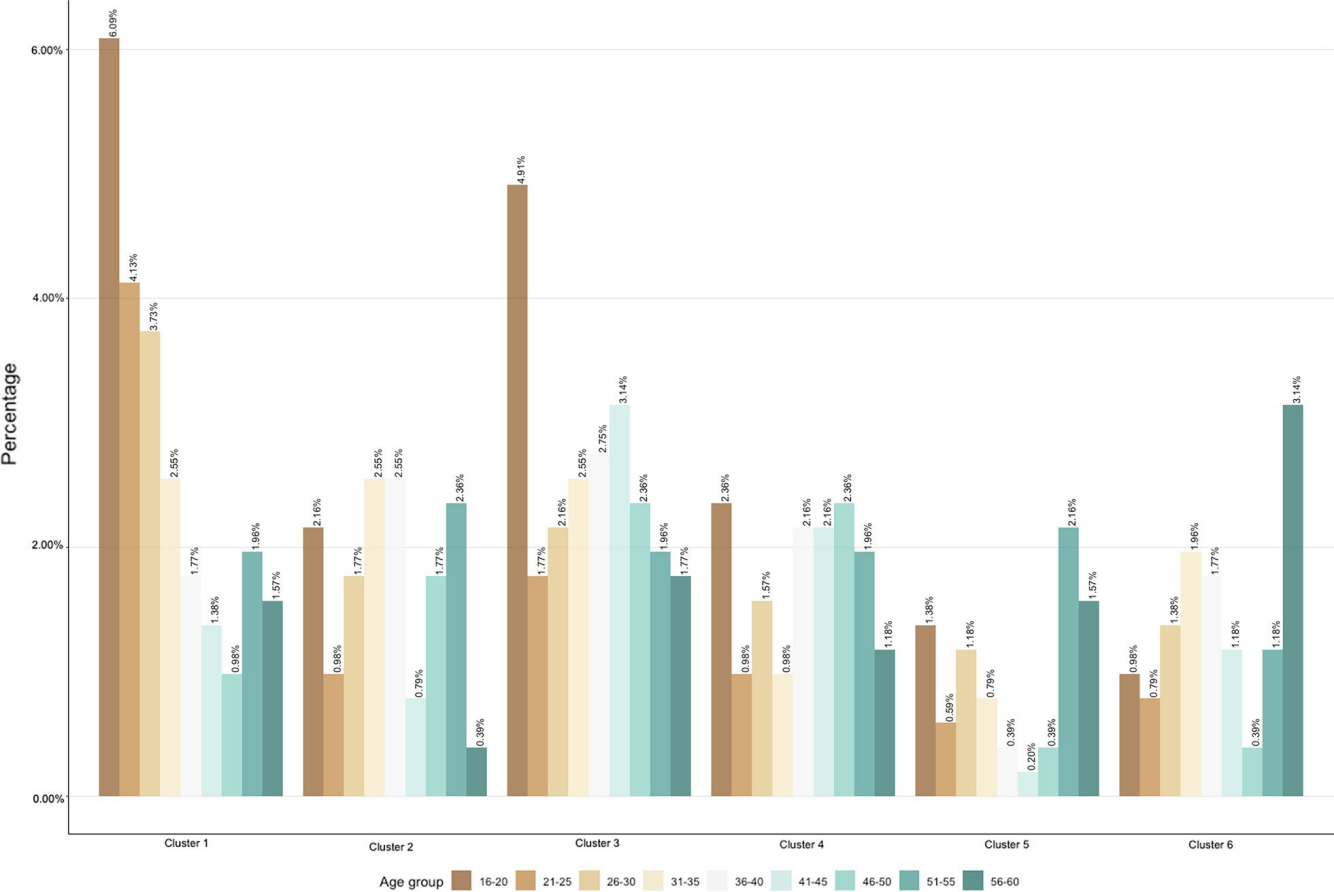
Age distribution of the samples collected in the 2009 and 2013 serosurveys by cluster. Age was classified as belonging to one of the following age-groups: 16–20; 21–25; 26–30; 31–35; 36–40; 41–45; 46–50; 51–55; 56–60 years

The age distribution of the samples in each cluster is presented in Fig. 3. The highest proportion of 16–20 years-old was observed in cluster 1 (25% of the subjects in the cluster are below 20 years of age). On the other hand, clusters 4 to 6 showed an older age distribution (35%, 48% and 37% of the subjects are above 46 years of age, respectively). Approximately two thirds (63% and 55%) of the subjects in clusters 2 and 3 were between 26 and 50 years of age. The age distribution of the samples in each cluster by year of sample collection is provided in Additional file 2: Figure S2.

**Fig. 4:**
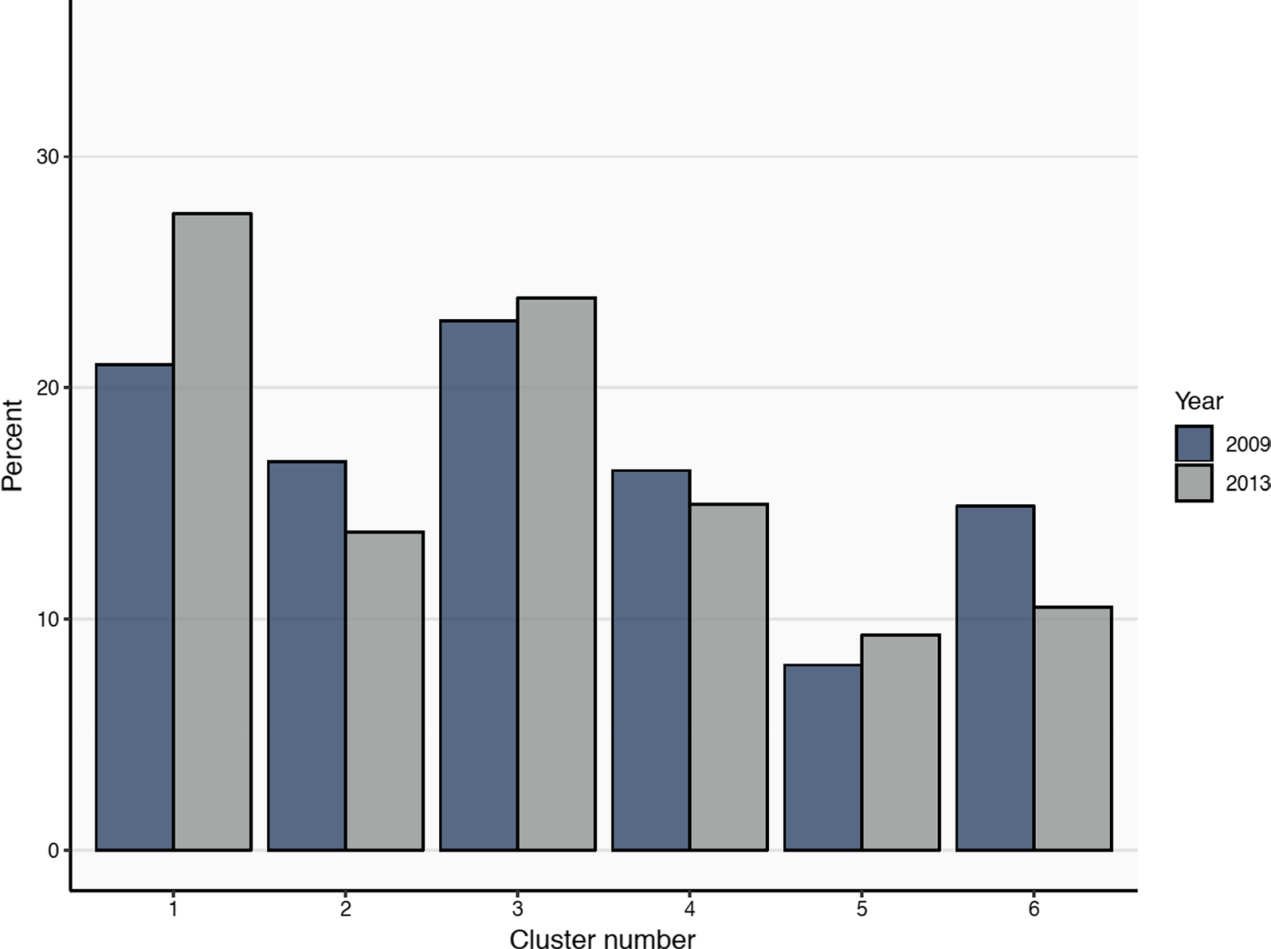
The percentage of subjects in cluster 1 to 6 by year of sample collection. Blue and grey colours represent samples collected in 2009 and 2013, respectively

Figure 4 shows a stratification of the PRNT_50_ titre profiles in each cluster by year of sample collection (2009 or 2013). Apart from clusters 1 and 6, where the number of individuals respectively increased and decreased by approximately 5% in 2013, we found that all other clusters comprised a stable (i.e. less than an interquartile range of percentage changes among the 6 clusters) and approximately equal number of subjects enrolled in 2009 and in 2013.

**Fig. 5:**
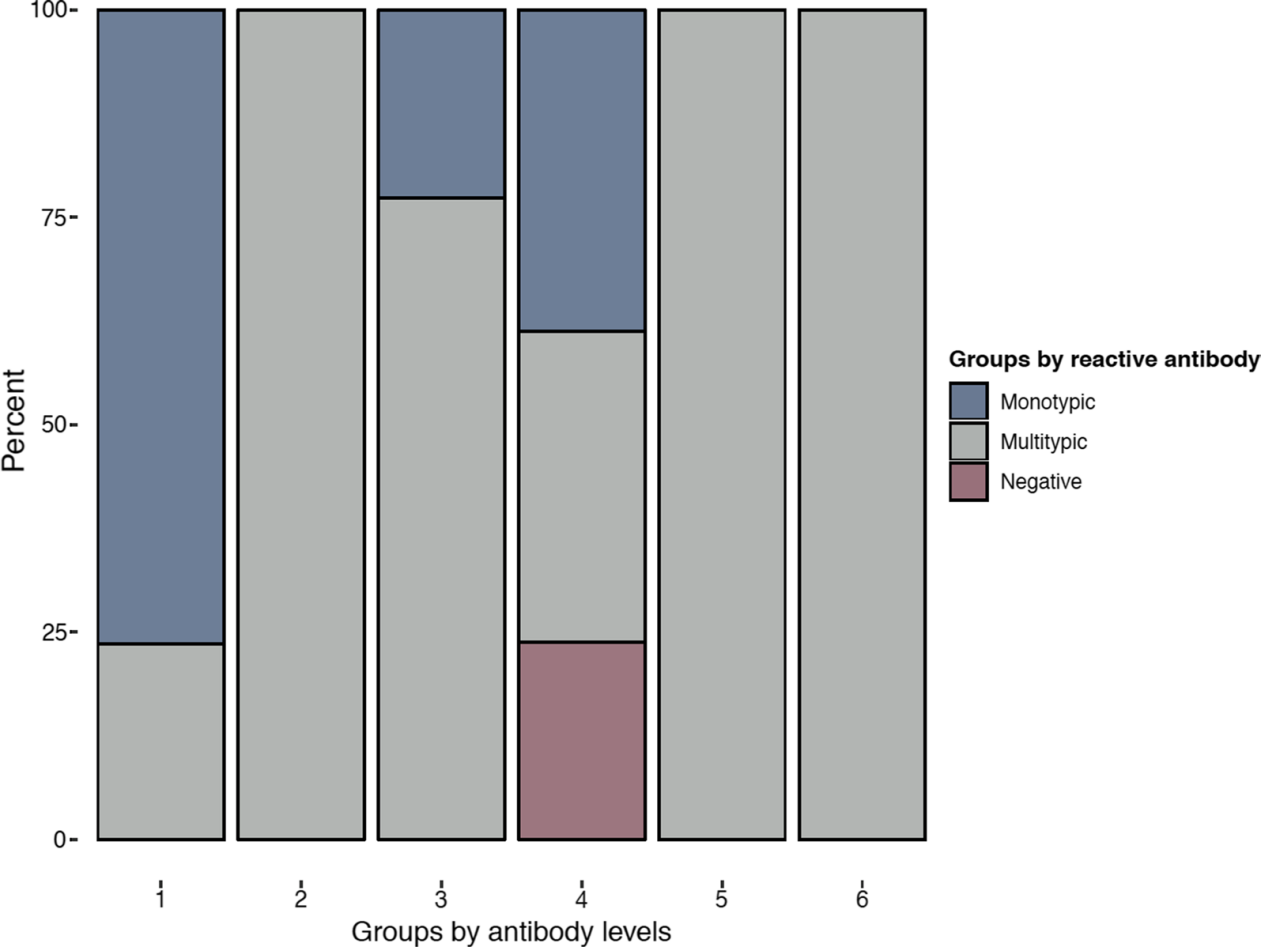
Comparison of the classification obtained using the threshold method to define exposure and the new classification obtained from cluster analysis. Seronegative subjects are characterised by all PRNT_50_ titres against DENV1-4 < 30; monotypic subjects display a single PRTN_50_ titre ≥ 30; multitypic subject is are defined as profiles with more than one PRNT_50_ titre ≥ 30

In Fig. 5 we show a comparison of the clusters obtained in our analysis (clusters 1–6) with the groups that would have been obtained using a cut-off threshold of PRNT_50_ titre at 30. Using the ‘threshold method’, which is generally adopted in the analysis of seroprevalence studies, samples were classified as seronegative (all PRNT_50_ titres against DENV1-4 < 30), monotypic (a single PRTN_50_ titre ≥ 30) and multitypic (more than one PRNT_50_ titre ≥ 30). Figure 5 shows that all monotypic patterns obtained using the ‘threshold method’ were assigned to clusters 1, 3 and 4; all multitypic patterns were assigned to clusters 2, 5 and 6; and all seronegative subjects were assigned to cluster 4.

In a sensitivity analysis, we explored the robustness of the results obtained using the aggregated (2009 and 2013) seroprevalence data with the data collected in 2009 and 2013 separately. The 2009 and 2013 seroprevalence studies respectively included 262 and 247 seropositive individuals with established PRNT_50_ titres. In the analyses on the data separated by year, we found that the agglomerative hierarchical method with 5 clusters and 6 clusters obtained the highest weighted rank aggregation of the three internal validating metrics for the 2009 and 2013 datasets, respectively. The top-five ranked clustering scenarios in each year and their internal validation metrics are presented in Additional file 1: Tables S3, S4. Additional file 2: Figure S3 shows the five clusters obtained from the analysis of the 2009 seropositive samples. Over three-fourths of the individuals in 2009 were in clusters characterised by DENV-2 dominant titres. In contrast, approximately half of individuals in 2013 were in clusters with DENV-1 dominant titres. These results are consistent with the dominance of DENV2 circulation in 2007–2009 and DENV1 circulation in 2013 [17]. All clusters obtained from the analysis of the 2013 PRNT_50_ data (Additional file 2: Figure S4) also show a remarkable similarity with the clusters obtained in the main analysis (Fig. 1). Using the Gower distance, the K-means method and the silhouette width to cluster the aggregated (2009 and 2013) PRNT_50_ data including the year of sampling as a covariate along with the PRNT_50_ variables, we obtained two single clusters that were entirely determined by the year of sample collection.

## Discussion

We found that the 509 dengue seropositive individuals from the seroprevalence surveys conducted in Singapore in 2009 and 2013 could be clustered into six groups on the basis of serotype-specific antibody levels. The six serotype-specific antibody profile patterns likely indicate similar histories of exposure to DENVs and similar risks of subsequent dengue infections.

The PRNT_50_ antibody titres of the vast majority of the individuals tested in the surveys were characterised by dominant or co-dominant DENV1 and/or DENV2 antibody levels, except for one small cluster (cluster 5 in Fig. 2) which displayed co-dominant DENV1, DENV2 and DENV3 titres. The dominant presence of antibodies against DENV1 and DENV2 indicates that these serotypes were the main circulating dengue serotypes in Singapore prior to 2009 and 2013, which is consistent with analyses of the surveillance data collected between 2003 and 2016 presented in Rajarethinam et al. [17].

Beyond providing information on the circulating serotypes, cluster analysis could be used in exploratory analyses to gain insight into the likely history of dengue exposure in the population. Following the studies of PRNT_50_ titre kinetics among children in Thailand, we refer to recent infections as infections that occurred less than a year before sampling and post-primary infection as secondary to quaternary infections [10, 11, 15]. We found that cluster 1, which is characterised by a single dominant PRNT_50_ titre against DENV2 (Fig. 2), identifies primary dengue infections according to the definitions proposed by Endy et al. [18] (PRNT_50_ titres ≥ 10 against more than one serotypes and ≥ 80 for the dominant serotype). The interpretation of cluster 1 as comprising primary dengue infections is supported by the young-age distribution of the subjects in this cluster compared to the other clusters (Fig. 3). Cluster 2 and 3 were characterised by co-dominant (2–3 log_10_) titres against DENV1 and DENV2, suggesting post-primary infections by DENV2 and DENV1, respectively. Inferring the order of the infecting serotypes is challenging as higher titres could be a result of boosting caused by infection with a heterologous serotype. The PRNT50 titres of clusters 5 and 6 are consistent with the observed titres of post-primary infections and the definition of multitypic infections (with DENV3 and DENV1 dominance, respectively) using the classical threshold method (Fig. 5). The relatively high PRNT50 titres against all serotypes observed in cluster 6 suggest that infections in this cluster occurred less than a year before sample collection [10, 11, 15]. Previous analyses of PRNT50 titres from clinical trial data [19] suggest that in post-primary infections DENV4 titres are on average half log_10_ lower than DENV1-3. The higher (more than 1 log_10_) difference in PRNT50 titres observed between DENV4 and DENV1-3 in cluster 6, along with the epidemiological evidence of DENV1-3 circulation in Singapore, suggests the heterologous and potentially cross-reactive nature of the antibody response against DENV4. This observation, together with the older age distribution observed in cluster 6 (relative to the age distribution of the other clusters) is consistent with the interpretation of this cluster as recent post-primary infections, which necessarily occur at an older age compared to the age of primary infections. The PRNT_50_ titre pattern of cluster 4 is in line with the one observed in recent primary infections where no dominance and relatively low antibody levels against all serotypes have been observed [10, 11, 15]. Dengue transmission in Singapore typically occurs from May to July. Sample collection occurred from December to February, hence infections occurred in May–July were between 6 and 10 months post-infection at sample collection, in line with our interpretation. Overall, 16% of the surveyed population was in cluster 4 (18% in 2009 and 11% in 2013) compared to 24% in cluster 1 (27% in 2009 and 30% in 2013), indicating the short-lived nature of heterologous cross-immunity.

The exploratory investigation of PRNT_50_ titres using cluster analysis also gives insight into the size of the population at potential risk of secondary, and hence severe, dengue infection. While individuals in clusters 2, 3, 5 and 6 showed post-primary-like PRNT_50_ titres and can thus be considered at a low risk of symptomatic infection, individuals in cluster 1, who showed a single dominant PRNT_50_ titre, might be vulnerable to antibody dependence enhancement (ADE). The relative proportions of samples collected in 2009 and 2013 forming clusters 6 and 1, respectively (with 5% higher proportion of subjects sampled in 2009 in cluster 6 and 5% higher proportion of subjects sampled in 2013 in cluster 1) suggest that DENV2 was the dominant serotype between 2009 and 2013. In clusters 1 and 4 (which were suggested as DENV-1 infections), the proportions of subjects between 36 and 55 years of age were relatively low in 2009 compared with the proportions observed in 2013. The considerable increase in the relative proportion of 36–55 years-old with DENV1 antibody titres from 2009 to 2013 is consistent and indicative of the occurrence of a DENV1 epidemic in 2013 [17]. The fact that DENV1 seroprevalence is high among older age-classes in 2013 is line with the relatively low force of infection of DENV1 in Singapore compared to other transmission settings and with the declining transmission intensity observed over the years and with population aging [20]. Similarly, the 10% increase in the proportion of the youngest age groups in cluster 1 between 2009 and 2013 (from 23 to 26.5%) is also indicative of the occurrence of a DENV2 epidemic between 2009 and 2013. In addition, in the analysis of the samples collected in 2013, the presence of cluster 5, which is characterised by multitypic patterns with DENV3 dominant titres, suggests that there was an increasing circulation of DENV3 between 2009 and 2013 in Singapore. These interpretations are consistent with the virus surveillance data presented in Rajarethinam et al. [17].

In this study, we presented an exploratory application of cluster analysis to classify seropositive individuals into groups with similar PRNT_50_ antibody patterns against specific dengue serotypes circulating in the Singaporean population in 2009–2013. The use of cluster analysis does not depend on pre-defined cut-off thresholds to define dengue exposure (which typically show substantial variations between laboratories) nor relies on a dichotomous classification of the PRNT_50_ titres into positive or negative results. While cluster analysis can provide qualitative information on the intensity of dengue transmission and detect the circulation of new serotypes, this framework does not allow to pin down how antibody titres change in time and the exact proportion of infections occurring in the clusters between seroprevalence surveys. In addition, the results of cluster analysis are sensitive to small changes in the data and to the specific clustering algorithm adopted for classification. In this analysis we used weighted rank aggregation using multiple internal validation metrics to ensure optimal clustering results. By study design, PRNT was performed on IgG seropositive samples and therefore the sampled population does not include the most recent primary infections that occurred around the sampling date (e.g. less than three weeks before the blood sample was taken) [21]. Moreover, because the samples tested by PRNT were randomly selected in an equal number within each age group, the age distribution of the samples used in this study is not representative of the actual seroprevalence in the Singaporean population.

In future work, the application of cluster analysis to PRNT_50_ titres with known infection outcome (e.g. from sero-epidemiological cohort studies) would allow to validate the clustering results and promote cluster analysis to classify the population into groups with different risks of developing dengue illness. Further analyses of sero-epidemiological data can provide useful information on the population-level risks of dengue epidemics and thus inform the development of public health policies, intervention strategies and outbreak response planning.

## Conclusions

We analysed the dengue serotype-specific PRNT tires of IgG seropositive participants enrolled in two serological surveys conducted in Singapore in 2009 and 2013 using cluster analysis. Cluster analysis is an unsupervised machine learning technique that was used to identify, within the sampled population, similar dengue antibody patterns which likely reflect similar infection histories. This exploratory technique, which does not depend on the use of cut off thresholds to define the serostatus, is a flexible tool to explore the immunity patterns of a population. We demonstrate that cluster analysis can provide new insights into the likely population-level histories of dengue exposure, existing levels of immunity and circulating serotypes which can help public health policy makers evaluate the risk of future epidemics and inform response planning.

## Supplementary information

**Additional file 1: Table S1.** Dengue virus strains used in the PRNT assay and variable codes used in the analysis. **Table S2.** Top 5 algorithms using weighted rank aggregation and the Dunn index, silhouette width, and adjusted connectivity as validating metrics from the aggregated analysis of the samples collected in 2009 and 2013. **Table S3.** Top 5 algorithms using weighted rank aggregation and the Dunn index, silhouette width, and adjusted connectivity as validating metrics from the analysis of the samples collectied in 2009. **Table S4.** Top 5 algorithms using weighted rank aggregation and the Dunn index, silhouette width and adjusted connectivity as validating metrics from the analysis of the samples collected in 2013.

**Additional file 2: Figure S1.** Correlation matrix of the eight variables in the data set (see Additional file 2: Table S1 for the variable definitions). The size and colour represent the correlation between any two variables, with larger circles representing higher correlation. **Figure S2.** Age distribution of the samples collected in the 2009 and 2013 serosurveys separately, by cluster. Age was classified as belonging to one of the following age-groups: 16–20; 21–25; 26–30; 31–35; 36–40; 41–45; 46–50; 51–55; 56–60 years. **Figure S3.** Median (bold line), interquantiles (box), range (vertical line) and outliers (points) of the log_10_ PRNT_50_ titres against DENV1-4 characterising the five clusters obtained with the agglomerative hierarchical clustering algorithm applied to the 2009 dataset. The colours blue, grey, green and brown represent DENV1, DENV2, DENV3 and DENV4, respectively. **Figure S4.** Median (bold line), interquantiles (box), range (vertical line) and outliers (points) of the log_10_ PRNT_50_ titres against DENV1-4 characterising the six clusters obtained with the agglomerative hierarchical clustering algorithm applied to the 2013 dataset. The colours blue, grey, green and brown represent DENV1, DENV2, DENV3 and DENV4, respectively.

## Data Availability

The data that support the findings of this study are available from the authors upon reasonable request and with permission of the National Environment Agency, Singapore.

## Abbreviations

ADE: antibody dependent enhancement
DALYs: disability-adjusted life years
DENV: dengue virus
PRNT: plaque reduction neutralisation test
SSE: sum of squared errors
EM: expectation maximization
