# Supplementary material for: Using cluster analysis to reconstruct dengue exposure patterns from cross-sectional serological studies in Singapore
Source: Parasit Vectors. 2020 Jan 17;13:32. doi: 10.1186/s13071-020-3898-5 (PMC6969465; doi:10.1186/s13071-020-3898-5)
Supplement: Supplementary file 1 — Additional file 1: Table S1. Dengue virus strains used in the PRNT assay and variable codes used in the analysis. Table S2. Top 5 algorithms using weighted rank aggregation and the Dunn index, silhouette width, and adjusted connectivity as validating metrics from the aggregated analysis of the samples collected in 2009 and 2013. Table S3. Top 5 algorithms using weighted rank aggregation and the Dunn index, silhouette width, and adjusted connectivity as validating metrics from the analysis of the samples collectied in 2009. Table S4. Top 5 algorithms using weighted rank aggregation and the Dunn index, silhouette width and adjusted connectivity as validating metrics from the analysis of the samples collected in 2013. [file 13071_2020_3898_MOESM1_ESM.docx]

**Additional file 1: Table S1.** Details of the dengue virus strains used in PRNT assay and variable codes used in this analysis

| Viral strains | Serotypes | Genotypes | Variable names |
| --- | --- | --- | --- |
| EHI0650Y08 | DENV1 | Genotype I | AD1 |
| EDEN203/05 | DENV1 | Genotype III | BD1 |
| EHI1170Y08 | DENV2 | Asian I | AD2 |
| EHI0866Y07 | DENV2 | Cosmopolitan Clade II | BD2 |
| EHI0040Y09 | DENV3 | Genotype I | AD3 |
| EDEN219/05 | DENV3 | Genotype III | BD3 |
| EHI462Y04 | DENV4 | Genotype I | AD4 |
| TCR310A129 | DENV4 | Genotype II | BD4 |

**Additional file 1: Table S2.** Algorithms achieved top 5 weighted rank aggregation and validating metric values from mixed 2009 and 2013 dataset

| Rank | Algorithm | Number of clusters | Dunn index | Sil. width | Connectivity | |
| --- | --- | --- | --- | --- | --- | --- |
| 1 | K-means | 6 | 0.0632 | 0.3200 | 161.2619 | |
| 2 | Divisive hierarchical | 5 | 0.0135 | 0.2760 | 174.2071 | |
| 3 | Aggromerative hierarchical | 7 | 0.0676 | 0.2761 | 143.2048 | |
| 4 | Aggromerative hierarchical | 8 | | 0.0723 | 0.2800 | 156.8306 |
| 5 | Divisive hierarchical | 6 | | 0.0449 | 0.3084 | 186.2381 |

**Additional file 1: Table S3.** Algorithms achieved top 5 weighted rank aggregation and validating metric values from 2009 dataset

| Rank | Algorithm | Number of clusters | Dunn index | Sil. width | Connectivity |
| --- | --- | --- | --- | --- | --- |
| 1 | Aggromerative hierarchical | 5 | 0.1187 | 0.2752 | 74.5210 |
| 2 | Aggromerative hierarchical | 4 | 0.0980 | 0.2827 | 54.4238 |
| 3 | Aggromerative hierarchical | 6 | 0.1007 | 0.2604 | 81.1714 |
| 4 | K-means | 4 | 0.0525 | 0.3451 | 76.2631 |
| 5 | Aggromerative hierarchical | 9 | 0.0955 | 0.2589 | 101.7317 |

**Additional file 1: Table S4.** Algorithms achieved top 5 weighted rank aggregation and validating metric values from 2013 dataset

| Rank | Algorithm | Number of clusters | Dunn index | Sil. width | Connectivity |
| --- | --- | --- | --- | --- | --- |
| 1 | Aggromerative hierarchical | 6 | 0.0870 | 0.3144 | 67.6992 |
| 2 | Aggromerative hierarchical | 7 | 0.0870 | 0.3048 | 78.4885 |
| 3 | K-means | 4 | 0.0755 | 0.3314 | 62.4778 |
| 4 | Divisive hierarchical | 7 | 0.0825 | 0.3244 | 106.1627 |
| 5 | K-means | 7 | 0.1008 | 0.3143 | 108.9187 |
