# Supplementary material for: Using cluster analysis to reconstruct dengue exposure patterns from cross-sectional serological studies in Singapore
Source: Parasit Vectors. 2020 Jan 17;13:32. doi: 10.1186/s13071-020-3898-5 (PMC6969465; doi:10.1186/s13071-020-3898-5)
Supplement: Supplementary file 2 — Additional file 2: Figure S1. Correlation matrix of the eight variables in the data set (see Additional file 2: Table S1 for the variable definitions). The size and colour represent the correlation between any two variables, with larger circles representing higher correlation. Figure S2. Age distribution of the samples collected in the 2009 and 2013 serosurveys separately, by cluster. Age was classified as belonging to one of the following age-groups: 16–20; 21–25; 26–30; 31–35; 36–40; 41–45; 46–50; 51–55; 56–60 years. Figure S3. Median (bold line), interquantiles (box), range (vertical line) and outliers (points) of the log10 PRNT50 titres against DENV1-4 characterising the five clusters obtained with the agglomerative hierarchical clustering algorithm applied to the 2009 dataset. The colours blue, grey, green and brown represent DENV1, DENV2, DENV3 and DENV4, respectively. Figure S4. Median (bold line), interquantiles (box), range (vertical line) and outliers (points) of the log10 PRNT50 titres against DENV1-4 characterising the six clusters obtained with the agglomerative hierarchical clustering algorithm applied to the 2013 dataset. The colours blue, grey, green and brown represent DENV1, DENV2, DENV3 and DENV4, respectively. [file 13071_2020_3898_MOESM2_ESM.docx]

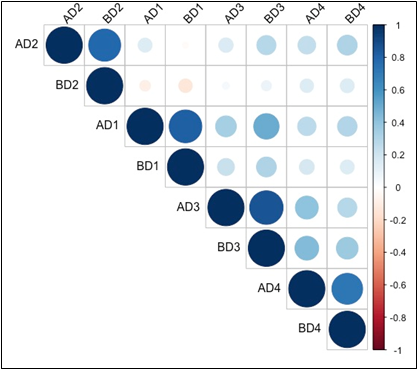


**Additional file 2: Figure S1.** Correlation matrix of theeight variables in the data set (see Additional file 2: Table S1 for the variable definitions). The size and colour represent the correlation between any two variables, with larger circles representing higher correlation.


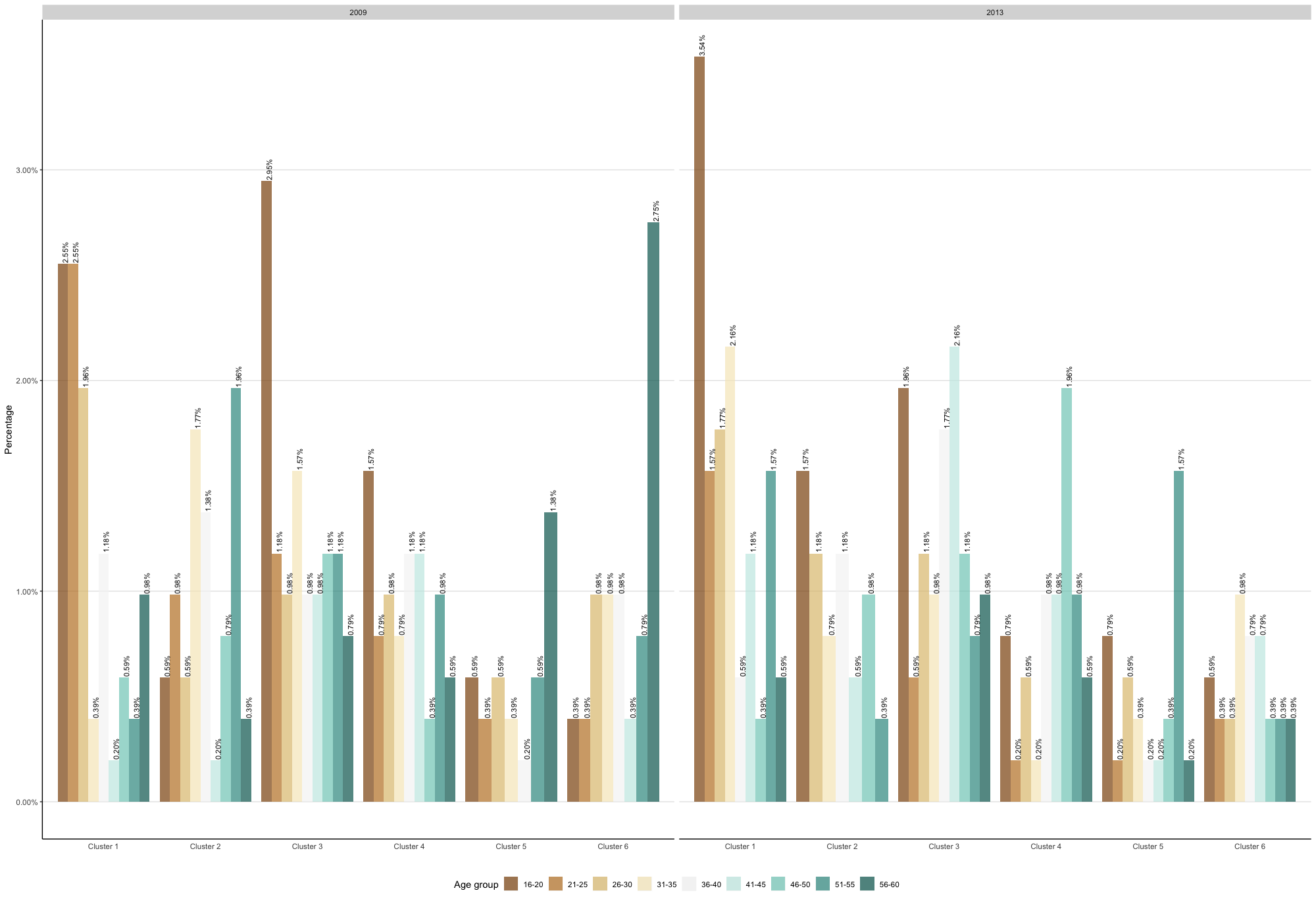


**Additional file 2: Figure S2.** Age distribution of the samples collected in the 2009 and 2013 serosurveys separately, by cluster. Age was classified as belonging to one of the following age-groups: 16–20; 21–25; 26–30; 31–35; 36–40; 41–45; 46–50; 51–55; 56–60 years.


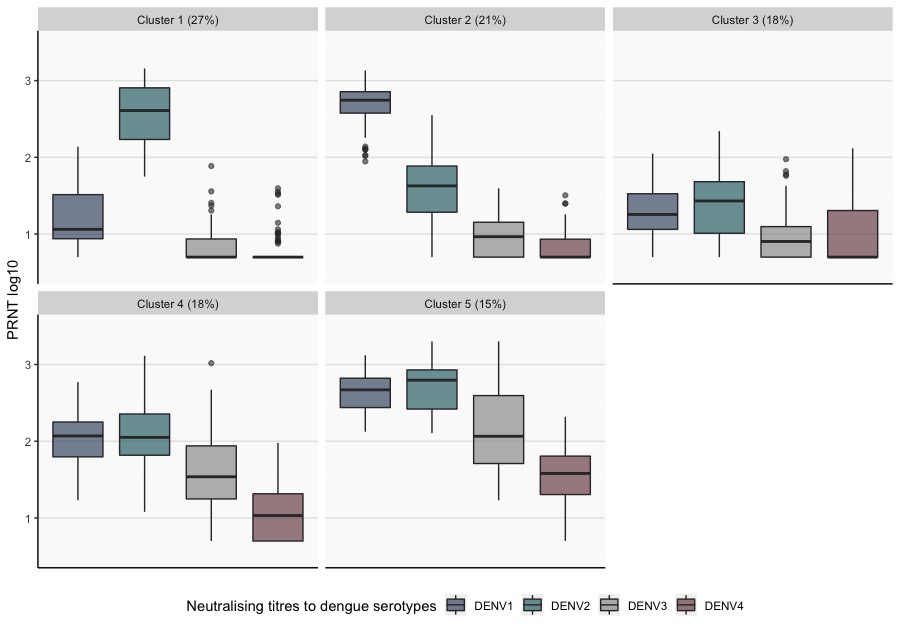


**Additional file 2: Figure S3.** Median (bold line), interquantiles (box), range (vertical line) and outliers (points) of the log_10_ PRNT_50_ titres against DENV1-4 characterising the five clusters obtained with an aggromerative algorithm from the 2009 dataset. The colours blue, grey, green and blown represent DENV1, DENV2, DENV3 and DENV4, respectively.


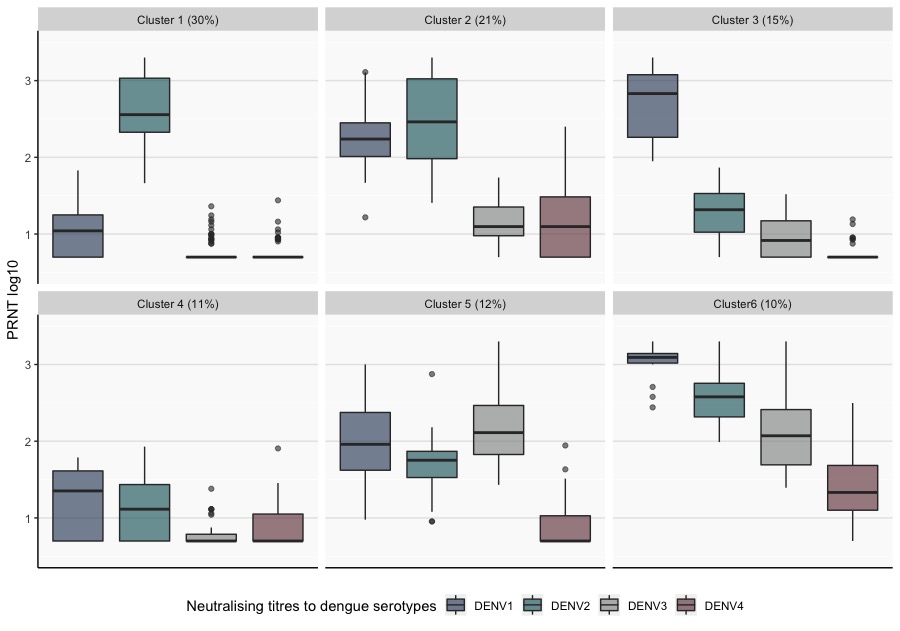


**Additional file 2: Figure S4.** Median (bold line), interquantiles (box), range (vertical line) and outliers (points) of the log_10_ PRNT_50_ titres against DENV1-4 characterising the six clusters obtained with an aggromerative algorithm from the 2013 dataset. The colours blue, grey, green and blown represent DENV1, DENV2, DENV3 and DENV4, respectively.
